# A Preliminary Assessment of HTST Processing on Donkey Milk

**DOI:** 10.3390/vetsci4040050

**Published:** 2017-10-09

**Authors:** Marzia Giribaldi, Sara Antoniazzi, Gian Marco Gariglio, Alessandra Coscia, Enrico Bertino, Laura Cavallarin

**Affiliations:** 1 Consiglio per la Ricerca in Agricoltura e L’analisi Dell’economia Agraria—Centro di Ricerca in Ingegneria e Trasformazioni Agroalimentari, 10135 Torino, Italy; 2 Consiglio Nazionale delle Ricerche—Istituto di Scienze delle Produzioni Alimentari, 10095 Grugliasco (To), Italy; sara.antoniazzi@ispa.cnr.it (S.A.); laura.cavallarin@ispa.cnr.it (L.C.); 3 Giada S.a.s., 10068 Villafranca P.te (To), Italy; gm.gariglio@giadapm.it; 4 Città della Salute e della Scienza—Struttura Complessa di Neonatologia, 10125 Torino, Italy; alessandra.coscia@unito.it (A.C.); enrico.bertino@unito.it (E.B.)

**Keywords:** donkey milk, holder, HTST, casein, lysozyme, pasteurization

## Abstract

Due to increasing attention from consumers on non-bovine milk types, and to the increase in the number of small dairy donkey farms in Italy, farmers require more advanced and reliable processing devices, in order to guarantee a safe product of high quality. To this aim, a new small-scale High-Temperature Short-Time (HTST) pasteurizer (72 °C for 15 s), prototyped by the authors, was tested on donkey milk. The efficacy of the HTST device was tested on raw donkey milk microflora by enumeration of total aerobic bacteria, Enterobacteriaceae and Bacillus cereus. The biochemical quality was assessed by determining the protein profile by monodimensional electrophoresis and by measuring lysozyme activity. The HTST apparatus was able to reduce the total bacteria count, and to completely eradicate Enterobacteriaceae. Bacillus cereus, when present, was decreased with low efficiency. Changes in the protein profile were observed in milk pasteurized in accordance with both processes, although HTST seemed to limit casein degradation. Lysozyme activity was not substantially affected in comparison to raw donkey milk. In conclusion, a tailored small-volume HTST device could be safely applied to pasteurize donkey milk in on-farm pasteurization processes on small dairy donkey farms.

## 1. Introduction

In recent years, attention towards donkey milk production and commercialization has been rising, mainly driven by its claimed difference in chemical and biochemical composition with respect to bovine milk, making it potentially suitable for consumption by specific consumers. Donkey milk was traditionally used in the past for its antibacterial and cosmetic properties [1,2], and its use as a bovine milk substitute has only been reported in the scientific literature recently. It’s only since the beginning of the century that the number of studies focusing on the protein fraction of donkey milk, and on its suitability for feeding children suffering from allergy to cow’s milk proteins (CMPA), has increased [3,4,5,6]. In addition, its immunomodulatory, anti-inflammatory, and anti-hypertensive properties have recently been studied both in in vitro and in vivo systems [7,8,9,10,11], with interesting perspectives. Very recently, a review paper on donkey milk was published, focusing on its past and future technological applications [12].

As a consequence, attention began to be paid to donkey milk transformation, in particular to its fermentation by lactic acid bacteria [13,14,15,16,17], to its storage [18,19,20], and to its sanitization [20,21,22]. Actually, the growing interest in donkey milk as a food for sensitive consumers, such as infants with allergies or immunocompromised elderly people, implies strict regulations for food safety [12]. Currently, mainly due to the small average size of farms producing and directly selling donkey milk, it is mainly provided raw, in compliance with European Community Regulation 853/2004. Regional and National guidelines specific to donkey dairy farming exist in Italy and Greece [12]. Holder pasteurization [21,22], spray drying [19,23], lyophilization [15,19] and, more recently, high-pressure processing [21,22,23,24] have been tested, and some of the resulting products can be found on the market, especially powders. Due to the increasing number of small dairy donkey farms in Italy, farmers need more sophisticated and reliable processing devices, in order to guarantee a safe product of high quality, and to increase their market share beyond the local region. These requirements include the possibility of pasteurizing milk on-farm. To date, the only available technologies for such uses are traditional devices that perform batch holder pasteurization (62.5 °C for 30 min) or batch high-temperature pasteurization (72 °C for 2 min). No analogous device to that commonly used in bovine milk industry for “fresh pasteurized milk” (High-Temperature Short-Time—HTST—pasteurization, 72 °C for 15 s), is available for processing low volumes, such as those required by dairy donkey farmers. 

We hereby present preliminary results of the application of a new small-scale continuous-flow HTST pasteurizer, patented by the authors, on donkey milk, in order to evaluate its efficacy in producing high-quality milk directly on-farm. The present research is aimed at reporting the efficacy of the new, low-volume, continuous flow HTST pasteurizer on donkey milk quality in terms of: (i) bacterial inactivation; (ii) preservation of protein profile; and (iii) lysozyme activity. The preliminary investigation indicates that this technology could represent an interesting opportunity for small dairy donkey farmers, providing a reduction of bacterial loads and a better preservation of the protein fraction of raw donkey milk.

## 2. Materials and Methods

Donkey milk was obtained from a small dairy donkey farm located in Groscavallo (To), which pooled the morning milking of the routinely milked animals (Martina Franca breed) and delivered 2 L of bottled refrigerated (4 °C) milk to our lab within 4 h. On arrival, milk was pooled (one starting pool) and mixed carefully in sterile Pyrex bottles, and three 200-mL control raw samples (referred as RDM—raw donkey milk—from now on) were sampled to be subjected to microbiological profiling, and then stored frozen (−20 °C) for less than one week until biochemical assays. From the remaining milk pool, four 250-mL aliquots were subjected to the tested pasteurization processes: two (HoDM—holder donkey milk) underwent holder pasteurization (63 °C for 30 min) in sterile Pyrex bottles in an agitated water bath; two (HTDM–HTST donkey milk) underwent HTST in the patented proprietary device (European Patent 2974603B1). To ensure an effective monitoring of time and temperature during the holder pasteurization, a third 250-mL donkey milk aliquot was equipped with a thermometer, and used as a probe; after 30 min from the reaching of 63 °C, the two HoDM bottles were cooled in ice, and then stored frozen (−20 °C for less than one week) until the biochemical assays. Meanwhile, the two HTDM aliquots were subjected to two separate HTST cycles (72 °C for 15 s) in the prototype, as described in a previous paper [25]. Between the two HTST pasteurizations, the prototype was washed in cleaning-in-place mode with 2% Amuchina (Angelini, Rome, Italy) for 10 min, and rinsed with tap water for 10 min. The HTDM was collected in sterile Pyrex bottles, and stored refrigerated until the morning after, when sampled to be subjected to microbiological profiling. The remaining HTDM was stored frozen until the biochemical assays.

The microbiological profiling of background microflora from the RDM and HTDM samples was performed by enumeration of total aerobic mesophilic viable count (Plate Count Agar PCA (Merck, Milan, Italy)—EN ISO 4833:2003-06 method), Enterobacteriaceae (Crystal-Violet Neutral-Red Bile Glucose Agar VRBGA (Merck)—AFNOR V08-054 method) and *Bacillus cereus* (Polymyxin pyruvate Egg yolk Mannitol Bromothymol blue Agar PEMBA (Biolife, Milan, Italy)—EN ISO 21871:2006 method).

The biochemical quality of all samples was monitored by analysis of some biochemical parameters, namely the protein profile and lysozyme activity, following the protocols already optimized in a previous study [26]. Briefly, the protein profile (in reducing conditions, 0.5 μL per sample) was visualized by monodimensional electrophoresis on a 10-well 12% Nu-PAGE^®^ precast gel (Life Technologies, Monza, Italy), with MES (2-(*N*-morpholino)ethanesulfonic acid, Life Technologies) as the running buffer, on a Novex Mini-cell (Life Technologies) at 200 V. One lane on each gel was loaded with 5 μL of Mark12^®^ Unstained Standard (Life Technologies). Two technical replicate gels were stained with Blue Coomassie colloidal stain, and protein band abundance was analyzed by QuantityOne software (Biorad, Milan, Italy). The identity of proteins contained in the single bands was putatively assigned by comparing the migration pattern with the donkey milk protein profile obtained from NuPAGE^®^ gels run in identical conditions, and identified by mass spectrometry. Lysozyme activity was tested in triplicate on 1:500 diluted samples using a fluorescence based kit (EnzChek Lysozyme Assay Kit, Life Technologies) following to the manufacturer’s instructions. 

Significant differences in the selected biochemical parameters were assessed by ANOVA at *p* ≤ 0.05, and classes of uniformity were grouped according to Tukey’s post-hoc test using KyPlot 2.0 software package (Kyens Lab Inc., Tokyo, Japan) 

## 3. Results

Table 1 reports the microbiological counts before and after HTST pasteurization. The starting RDM sample presented very low counts, consistent with reporting by other authors [27,28,29]. The new HTST device was shown to reduce Enterobacteriaceae counts to undetectable level, while total bacteria and *Bacillus cereus* were decreased, leaving very few colonies in milk (Table 1). 

**Figure 1:**
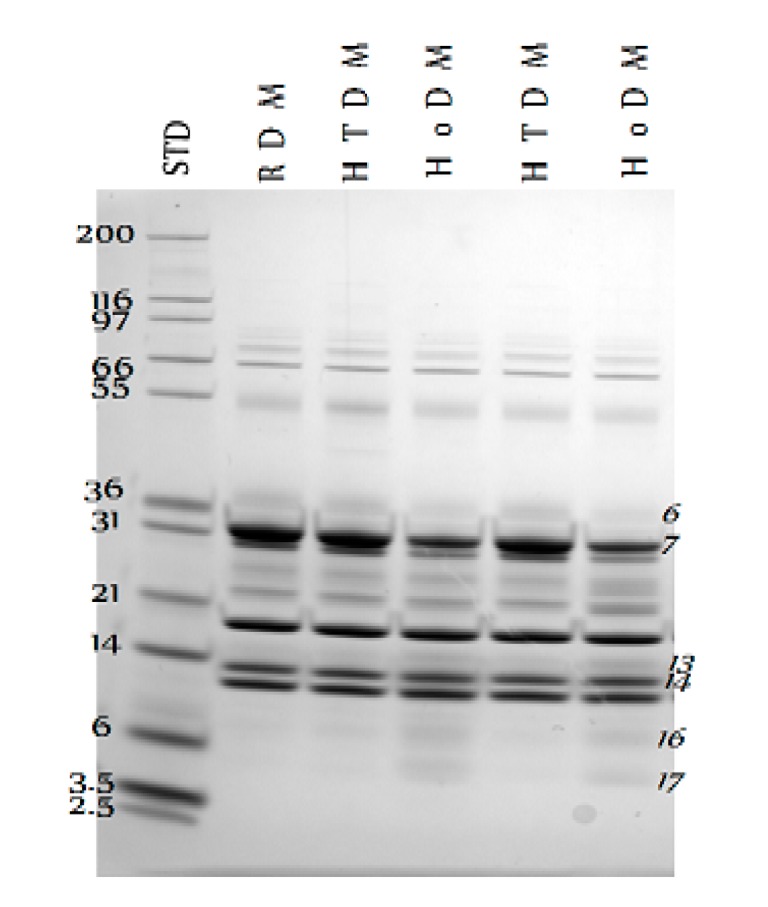
NuPAGE^®^ Protein profile of donkey milk before (RDM—raw donkey milk) and after HTST (HTDM—HTST donkey milk) and Holder (HoDM—holder donkey milk) pasteurization. STD: Mark12^®^ Unstained Protein Standard (Life Technologies), KDa. Numbers on the right indicate significantly different protein bands.

Changes in the protein profile were observed in the milk pasteurized according to both processes (Figure 1). In detail, HTST seems to preserve the protein profile more than the traditional holder method, limiting the degradation of some bands owing to the casein fraction (labeled as black squares in Figure 1), which results in an increase in lower molecular weight bands (labeled as black ellipses in Figure 1). Results from image analysis are reported in Table 2: bands 6 and 7 were significantly decreased in HoDM, while bands 13, 14, 16 and 17 were increased.

As for lysozyme activity, a slight, not significant, decrease (5.5%) was found (Table 3), thus indicating that both methods are suitable for preserving almost all the antibacterial potential associated with this enzyme, in accordance with faint variation of lysozyme protein band in electrophoresis (band 14, small increase after pasteurization).

## 4. Discussion

The present preliminary results represent the first contribution to the potential application of HTST pasteurization, which is commonly used in the bovine milk industry for “fresh pasteurized milk”, to small-/medium-sized dairy donkey farms, which have increased in number in Italy in the last decade. Due to the relatively low requirements of donkey farming in terms of space, animal robustness, and high economic value, donkey farming is considered an interesting opportunity for small farmers in rural environments, such as mountain farmers. This peculiarity could therefore complicate its delivery to interested consumers, and it would be in the interests of producers to extend the shelf life of the product. The mean production of this type of dairy donkey farm is relatively low (less than 30 L/day) and, to date, the available pasteurization technology for such use has been limited to traditional devices that perform batch holder pasteurization (62.5 °C for 30 min) or batch high-temperature pasteurization (72 °C for 2 min). We, therefore, performed the present preliminary feasibility assessment using a prototyped patented device specifically designed to continuously pasteurize low volumes of milk by HTST (72 °C for 15 s), in order to guarantee its safety, extend its potential shelf-life (which is currently fixed at 3 days for raw milk [21]), and to preserve its nutritional and biochemical quality more than traditional devices.

Bacteriological analyses on the HTST-pasteurized donkey milk revealed that the prototype was able to reduce both total aerobic counts (>1 log reduction) and *Bacillus cereus*, and to eliminate Enterobacteriaceae. Starting bacteriological counts were very low, but efficacy in reduction of potentially damaging microorganisms is interesting, and comparable to what has previously been reported in the literature for holder pasteurization [13,21] and high-pressure processing [21,24]. This evidence should be confirmed on highly contaminated donkey milk batches. Moreover, the potential shelf-life extension should be evaluated on refrigerated HTST-pasteurized donkey milk [21]. As for biochemical and nutritional retention of analyzed components, HTST seems to be as effective as Holder pasteurization in retaining lysozyme activity, confirming observations from other authors that temperatures higher than 80 °C and longer processing times are required to observe substantial lysozyme activity reductions [18,19,20,30]. Despite a similar method of analysis being used, lysozyme activity in the starting sample was much higher than what was reported in the Amianta breed by other authors [27,29], but was consistent with values reported on the same farm in a previous survey [26], and with results from Greek donkeys [31]. The differences have been explained by endogenous and physiological factors (breed and lactation period), or as being related to sample processing [27,29]. Regardless of the reason, the high lysozyme activity of the starting sample may contribute to the very low bacterial counts of the raw sample.

Nevertheless, visible changes in the protein profile of donkey milk were observed between different pasteurization treatments. In particular, the casein fraction seems to be substantially degraded by holder pasteurization, while HTST preserved the raw milk casein fraction. This is in good agreement with the observation by other authors [18,19], reporting a lower thermostability of the casein fraction of donkey milk with respect to lysozyme. Some authors reported that flocculation and sedimentation phenomena had been observed in donkey milk when subjected to High-Pressure Processing [21,24], which were tentatively attributed to the higher whey protein content in donkey in comparison to cow’s milk. Our results indicate that the casein fraction may contribute to these phenomena, because of its instability and susceptibility to degradation. Further investigation of the issue is needed, also due to its potential implications for donkey milk tolerability for sensitive consumers. Comparison of our results with that from Ozturkoglu Budak [20], which treated donkey milk at 75 °C for 2 min in a batch-simulated HTST process, also indicates that continuous-flow HTST pasteurization with the patented prototype ensures a higher retention of other whey proteins, such as beta lactoglobulin and lactoferrin.

## 5. Conclusions

In conclusion, the new HTST device was able to be safely applied to pasteurizing donkey milk, despite the low volumes involved in the standard routine of small dairy donkey farms. Moreover, the HTST device seems to ensure a higher retention of some protein fractions, such as caseins. The present findings should be considered preliminary, and need to be confirmed on other milk batches, since only one donkey milk pool was used. Further tailored device prototyping could contribute both to ensuring consumer safety—in particular when used for cow’s milk protein-allergic infants—and to the exploitation of farmers’ markets.

## Figures and Tables

**Table 1:** Microbiological counts (total aerobic— Plate Count Agar (PCA), Enterobacteriaceae—Crystal-Violet Neutral-Red Bile Glucose Agar (VRBGA) and *Bacillus cereus*—Polymyxin pyruvate Egg yolk Mannitol Bromothymol blue Agar (PEMBA) counts) of donkey milk before (RDM—raw donkey milk) and after (HTDM—HTST donkey milk) High Temperature Short Time (HTST) pasteurization. Counts are expressed as mean ± standard deviation Colon Forming Units per mL (N = 3 for RDM; N = 6 for HTDM).

	PCA	VRBGA	PEMBA
RDM	99 ± 5	20 ± 6	7 ± 3
HTDM	8 ± 3	-	1 ± 1

**Table 2:** Image analysis derived relative intensity data of protein bands in donkey milk before (RDM—raw donkey milk) and after HTST (HTDM – HTST donkey milk) and Holder (HoDM—holder donkey milk) pasteurization. SD: standard deviations (N = 4). Letters indicate post-hoc classes according to Tukey’s test when variation in band intensity was above 25%. NS: not significant. * *p* < 0.05; ** *p* < 0.01; *** *p* < 0.001.

RDM		HoDM		HTDM		
*Relative Intensity*	*SD*	*Relative Intensity*	*SD*	*Relative Intensity*	*SD*	*Significance*
0.848	0.009	0.775	0.094	0.947	0.113	NS
0.788	0.100	0.969	0.169	0.851	0.054	NS
1.91	0.117	1.92	0.219	1.93	0.052	NS
1.92	0.016	1.84	0.044	1.86	0.134	NS
2.98	0.644	3.14	0.178	3.34	0.190	NS
3.66 A	0.087	2.74 B	0.555	3.08 AB	0.483	*
5.01 B	0.068	4.11 A	0.263	5.46 C	0.226	***
2.33	0.056	2.20	0.113	2.53	0.112	*
2.60	0.097	2.42	0.347	2.26	0.210	NS
1.16	0.161	1.38	0.245	1.36	0.143	NS
3.37	0.036	3.39	0.66	2.88	0.122	NS
4.93	0.384	4.53	0.397	4.95	0.051	NS
1.91 A	0.025	2.87 B	0.164	2.12 A	0.286	*
3.12 A	0.291	3.95 B	0.225	3.62 AB	0.359	*
3.60	0.132	3.92	0.157	4.08	0.265	*
2.07 A	0.681	3.40 B	0.615	2.27 A	0.337	*
2.49 AB	0.631	3.48 B	0.581	2.20 A	0.528	*

**Table 3:** Lysozyme activity in donkey milk before (RDM—raw donkey milk) and after HTST (HTDM—HTST donkey milk) and Holder (HoDM—holder donkey milk) pasteurization. Means are expressed as Activity units per µL. SD: standard deviations (N = 3 for RDM; N = 6 for HTDM and HoDM).

Sample	Enzyme Activity	SD
RDM	86.7	6.9
HoDM	81.9	3.5
HTDM	81.0	0.7
